# Identifying risk factors for chronic kidney disease stage 3 in adults with acquired solitary kidney from unilateral nephrectomy: a retrospective cohort study

**DOI:** 10.1186/s12882-020-02059-2

**Published:** 2020-09-14

**Authors:** Wen-Jun Zhang, Zi-Yi Wang, Wei-Xing Zhou, Ning-Qiang Yang, Ya Wang, Ya Tang, Xiao-Chun Zhou, Jie-Cao Dao, Yan-Ru Ma, Yan-Ping He, Xiao-Ling Wang, Wen-Ge Wang, Li Yang

**Affiliations:** 1 Department of Nephrology, Lan Zhou University Second Hospital, Lanzhou, 730030 China; 2 Department of Nephrology, GanSu University of Chinese Medicine, Lanzhou, 730030 China; 3 Department of Urology Surgery, Lan Zhou University Second Hospital, 82 Cui Ying Gate, Lanzhou, 730030 China

**Keywords:** Acquired solitary kidney, Chronic kidney disease, Risk factor

## Abstract

**Background:**

We aimed to examine the risk factors for chronic kidney disease (CKD) stage 3 among adults with ASK from unilateral nephrectomy.

**Methods:**

We retrospectively collected data from adult patients with ASK between January, 2009 and January, 2019, identified from a tertiary hospital in China. The clinical data were compared between patients who developed CKD stage 3 and those who did not develop CKD stage 3 during follow-up.

**Results:**

In total, 172 patients with ASK (110 men; median 58.0 years) were enrolled, with a median follow-up duration of 5.0 years. During follow-up, 91 (52.9%) and 24 (14.0%) patients developed CKD stage 3 and end-stage renal disease, respectively. Multiple regression analyses showed that age (odds ratio [OR] 1.076, 95% confidence interval [CI] 1.039–1.115, *p* < 0.001), diabetes (OR 4.401, 95% CI 1.693–11.44, *p* = 0.002), hyperuricemia (OR 2.733, 95% CI 1.104–6.764, *p* = 0.03), a history of cardiovascular disease (CVD) (OR 5.583, 95% CI 1.884–18.068, *p* = 0.002), and ASK due to renal tuberculosis (OR 8.816, 95% CI 2.92–26.62, *p* < 0.001) were independent risk factors for developing CKD stage 3 among patients with ASK.

**Conclusions:**

Regular follow-up of renal function is needed among adult patients with ASK. Optimal management of diabetes, hyperuricemia, and CVD may reduce their risk of CKD stage 3, especially among those that undergo unilateral nephrectomy for renal tuberculosis.

## Background

The origin of solitary kidney can be congenital or acquired. Congenital solitary kidney (CSK) is mainly due to unilateral renal agenesis, while acquired solitary kidney (ASK) after unilateral radical nephrectomy in adults is frequently the result of treatment for renal tumors, severe parenchymal renal infections, renal trauma, and kidney donation. Patients with a solitary kidney have by definition renal mass reduction, which leads to functional and structural changes among the remaining glomeruli, followed by compensatory glomerular hypertension, hyperfiltration, and hypertrophy [1]^.^ These adaptive changes may have short-term benefits, but the long-term effect can be harmful to the solitary kidney [2]^.^ Although the risk of impaired renal function in the solitary kidney has been studied before, available reports predominantly focus on patients with CSK or those who are kidney donors [3–7].

Existing studies consistently indicate an increased long-term risk of end-stage renal disease (ESRD) and higher mortality among live kidney donors. A population-based study showed that the estimated risk of developing ESRD 15 years after donation was 30.8 and 3.9 per 10,000 patients among live kidney donors and matched healthy persons, respectively, and the estimated lifetime risk of developing ESRD was 90 and 14 per 10,000, respectively [6]. A meta-analysis of seven cohorts based on the general population also identified 3.5–5.3 times higher risk for developing ESRD among live kidney donors compared to age-matched controls [7]. However, very few studies address the renal outcomes among patients with ASK due to other causes, which are more commonly encountered in clinical practice following unilateral radical nephrectomy for renal tumors, renal tuberculosis, or renal trauma.

The current study aimed to identify important risk factors associated with the development of CKD stage 3 among adults with ASK and to examine the renal outcomes. The information gleaned from this study will help provide optimal management for patients with ASK through protecting their renal function.

## Methods

### Study population and study design

This retrospective study was conducted in the Lanzhou University Second Hospital, a 3000-bed university-affiliated and tertiary care referral hospital in GanSu, China. We identified patients with an international classification of disease – 10th version (ICD-10) code of Z90.5 (solitary kidney) or Q60.0 (unilateral kidney deficiency) between January 2009 and January 2019 from the electronic medical records, and included those older than 18 years old who underwent unilateral nephrectomy. The exclusion criteria consisted of individuals with abnormal proteinuria and eGFR greater than 60 ml/min/1.73 m^2^ or a history of CKD before nephrectomy, and those who were pregnant. We also collected their clinical and laboratory data, including age, gender, smoking status, history of cardiovascular diseases, years after nephrectomy, body mass index (BMI), blood pressure (BP), comorbidities such as hypertension and diabetes, serum creatinine, urea, uric acid, fasting glucose, total cholesterol (TC), low-density lipoprotein (LDL) cholesterol, high-density lipoprotein (HDL) cholesterol, triglycerides, and spot urine and/or 24-h urine protein levels. Cardiovascular diseases included myocardial infarction, stroke, transient ischemic attack, intermittent claudication, prior revascularization, and/or amputation due to peripheral vascular disease. BMI was calculated based on their weight in kilograms divided by the square values of height in meters. Serum creatinine and uric acid concentrations were measured by the picric kinetic method and the enzymatic method, respectively. Their estimated glomerular filtration rate (eGFR) was calculated using the Chronic Kidney Disease Epidemiology Collaboration (CKD-EPI) equation [8]. This study was approved by the Ethics Committee of Lanzhou University Second Hospital (No. 2019A-154).

### Definitions of variables and outcomes

Hypertension was defined as having a systolic and/or diastolic BP ≥ 140 and 90 mmHg, respectively, a history of hypertension, or the use of any antihypertensive medication. Diabetes was defined as having a fasting glucose ≥7.0 mmol/L, a history of diabetes, or the use of any antidiabetic medication. Hyperuricemia was defined as having a serum uric acid ≥420 μmol/L (if male) or 360 μmol/L (if female). Dyslipidemia was defined as having TC ≥ 6.2 mmol/L, LDL cholesterol ≥4.1 mmol/L, HDL cholesterol < 1.0 mmol/L, and/or triglycerides ≥2.3 mmol/L. Obesity was defined as having a BMI ≥ 25 kg/m^2^ according to the Asian-specific criteria [9]. Current smokers were defined as patients who were active smokers at the time of the nephrectomy or had stopped smoking for less than or equal to 1 year before nephrectomy. Patients were regarded as having proteinuria if their 24-h urine protein level exceeded 150 mg per day or if their routine urinalysis for protein was positive. Three or more red blood cells observed under a high-power field were considered hematuria. The development of CKD stage 3 was designated as the study outcome. CKD was defined as having an eGFR < 60 mL/min/1.73 m^2^ and/or the presence of proteinuria and/or the presence of hematuria in two or more tests at the end of follow-up. The staging of CKD was performed according to the 2012 Clinical Practice Guidelines for the Global Prognosis of Kidney Disease. ESRD was defined as having an eGFR less than 15 mL/min/1.73 m^2^, the initiation of chronic dialysis, or receiving a kidney transplant.

### Statistical analysis

All statistical analyses were performed using SPSS version 25.0 (SPSS Inc. Chicago, IL, USA). To identify independent risk factors for the development of CKD stage 3, we compared clinical parameters between patients who developed CKD stage 3 and those who did not develop CKD stage 3 during follow-up. Continuous variables were expressed as means ± standard deviation and categorical variables as numbers with percentages. A Shapiro-Wilk test was used to analyze the normality of the data distribution. Non-normally distributed data were expressed as medians with interquartile ranges. We used the Student’s t-test or the Mann-Whitney test and the Chi-square test or the Fisher’s exact test to compare continuous and categorical variables, respectively. We used the Pearson’s parametric test or the Spearman non-parametric test for correlation analyses. We used the logistic regression analyses to identify independent factors associated with the development of CKD stage 3. A *p* value < 0.05 was considered statistically significant in all analyses.

## Results

### Demographic characteristics

We summarized the clinical characteristics of the patients in Table 1. We included 172 patients with ASK (110 men), with a median age of 58.0 years. The median duration after unilateral nephrectomy among the included patients was 5.0 years. Ninety-one (52.9%) patients developed CKD stage 3 and 24 (14.0%) patients developed ESRD.

**Table 1:** Baseline Clinical Characteristics of Adults with Acquired Solitary Kidney

Parameter	Value
No	172
Sex (M/F)	110/62
Age (years)	58.0 (21.0–73.0)
Current smoker	40 (23.3%)
History of cardiovascular disease	39 (22.7%)
Cause of ASK	
Tumors	72 (41.9%)
Tuberculosis	50 (29.1%)
Trauma	7 (4.1%)
Other	16 (9.3%)
Unknown	27 (15.6%)
Serum creatinine (*μmol*/*L*)	83.6 (71.4, 106.0)
eGFR (ml/min/1.73 m^2^)	92.4 ± 25.0
Comorbidities	
Hypertension (*N* = 168)	88 (52.4%)
Obesity (*N* = 155)	52 (33.5%)
Diabetes (*N* = 162)	44 (27.2%)
Hyperuricemia (*N* = 169)	59 (34.9%)
Dyslipidemia (*N* = 146)	21 (14.4%)

Note: Results are presented as numbers (percentages), medians (interquartile range), or medians (standard deviation); *eGFR* estimated glomerular filtration rate, *ASK* acquired solitary kidney, *Scr* Serum creatinine

**Fig. 1:**
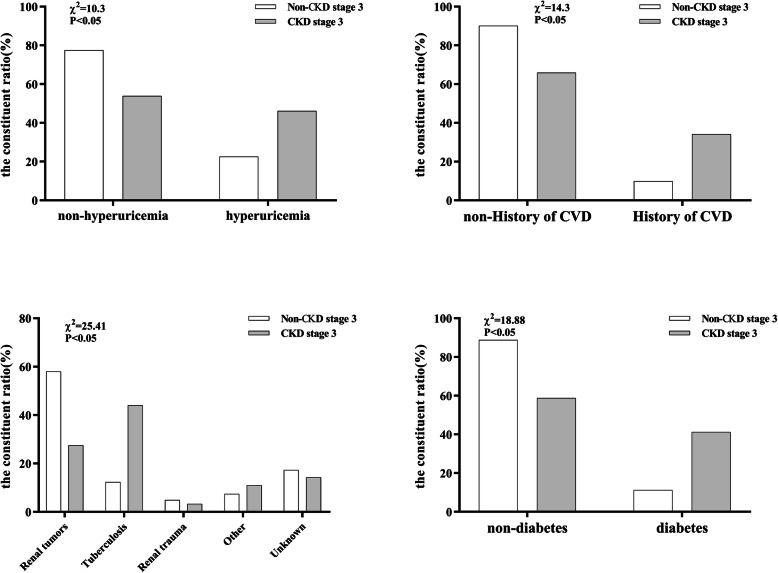
The proportion of patients with acquired solitary kidney that received nephrectomy for renal tuberculosis was significantly higher than those with other conditions among the patients who developed CKD stage 3. The prevalence of diabetes, hyperuricemia, and history of CVD was also higher among those that developed CKD stage 3 than among those that did not

The main reasons for nephrectomy were renal tumors (*n* = 72; 41.9%) and renal tuberculosis (*n* = 50; 29.1%). The proportion of patients with ASK that received nephrectomy for renal tuberculosis was significantly higher than those with other conditions among the patients who developed CKD stage 3 (Fig. 1). Parameters for patients in different CKD stages are shown in Table 2.

**Table 2:** Parameters for patients in different CKD stages

		CKD 1–2	CKD 3–4	ESRD
		*n* = 21	*n* = 67	*n* = 24
Sex	Male	12 (17.6%)	42 (61.8%)	14 (20.6%)
	Female	9 (20.5%)	25 (56.8%)	10 (22.7%)
Age (years)		52 (42–62)	62 (56–73)	56.5 (47–63.8)
Duration after nephrectomy (years)		4 (2.5–10.5)	5 (1–12)	12 (5–19.5)
Serum creatinine (*μmol*/*L*)		81 (68.5–99)	127 (112–150.3)	658.5 (475.3–996.2)
eGFR (ml/min/1.73 m^2^)		85.5 ± 14.9	46.7 ± 9.2	6.3 ± 2.5
Proteinuria	No	12 (23.1%)	37 (71.2%)	3 (5.7%)
	Yes	9 (20%)	24 (53.3%)	12 (26.7%)
Hematuria	No	8 (10.1%)	53 (67.1%)	18 (22.8%)
	Yes	13 (39.4%)	14 (42.4%)	6 (18.2%)
Kidney location	Left	8 (14.0%)	37 (64.9%)	12 (21.1%)
	Right	13 (23.6%)	31 (56.4%)	11 (20%)
Cause of ASK	Tumors	11 (30.6%)	22 (61.1%)	3 (8.3%)
	Tuberculosis	6 (13%)	29 (63%)	11 (23.9%)
	Trauma	1 (25%)	1 (25%)	2 (50%)
	Other	1 (9.1%)	5 (45.5%)	5 (45.5%)
	Unknown	2 (13.3%)	10 (66.7%)	3 (20%)
Current smoker	No	17 (19.8%)	50 (58.1%)	19 (22.1%)
	Yes	4 (15.4%)	17 (65.4%)	5 (19.2%)
History of CVD	No	18 (23.1%)	46 (59%)	14 (17.9%)
	Yes	3 (8.8%)	21 (61.8%)	10 (29.4%)
Hypertension	No	9 (19.1%)	30 (63.8%)	8 (17%)
	Yes	11 (18%)	35 (57.4%)	15 (24.6%)
Obesity	No	12 (18.5%)	36 (55.4%)	22 (26.2%)
	Yes	5 (14.3%)	24 (68.6%)	6 (17.1%)
Diabetes	No	20 (28.6%)	35 (50%)	15 (21.4%)
	Yes	1 (2.8%)	28 (77.8%)	7 (19.4%)
Hyperuricemia	No	16 (25%)	38 (59.4%)	10 (15.6%)
	Yes	4 (8.9%)	27 (60%)	14 (31.1%)
Dyslipidemia	No	14 (15.1%)	58 (62.4%)	21 (22.5%)
	Yes	2 (18.2%)	9 (81.8%)	0 (0%)

Note: Results are presented as numbers (percentages), medians (interquartile range), or medians (standard deviation); *eGFR* estimated glomerular filtration rate, *ASK* acquired solitary kidney, *CVD* cardiovascular disease

### Risk factors for developing CKD stage 3

**Fig. 2:**
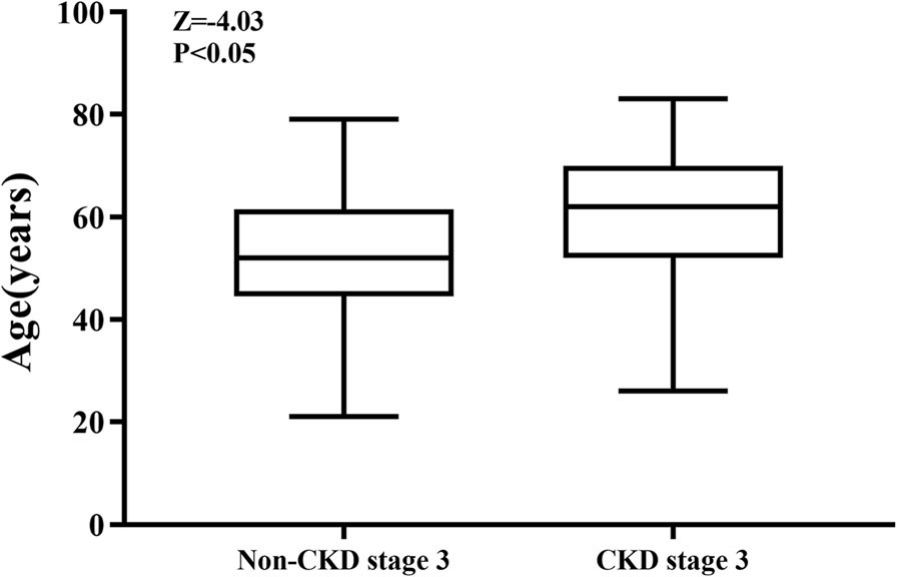
Box-whisker plot showing that patients who developed CKD stage 3 were older than those who did not develop CKD stage 3

**Fig. 3:**
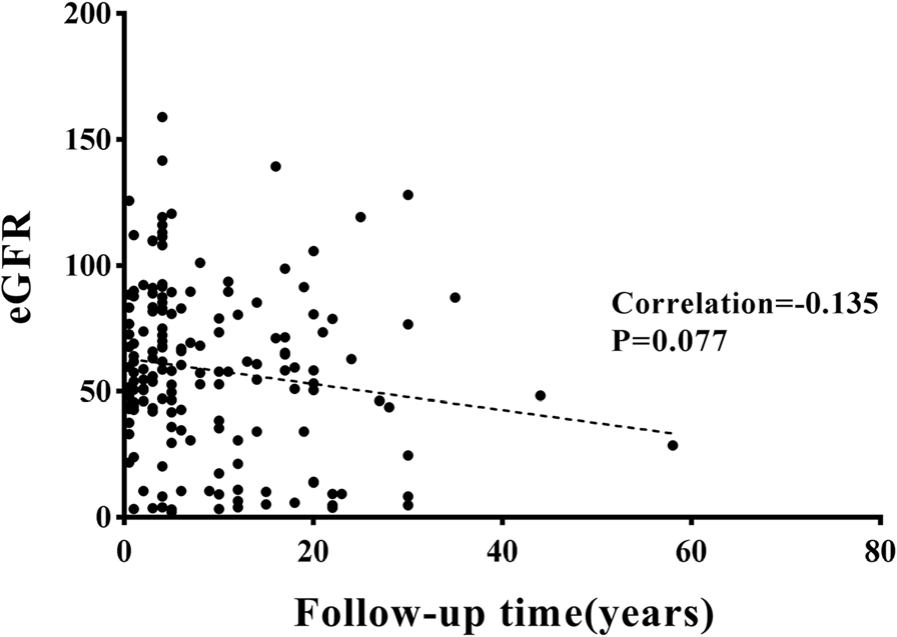
A scatter plot showing a negative correlation between eGFR and the duration after nephrectomy, but the correlation did not reach statistical significance (*r* = −0.135, *p* = 0.077)

In univariate analyses, we found significant differences between patients who developed CKD stage 3 and those who did not develop CKD stage 3 with regard to their age, history of CVD, current smoking status, the prevalence of diabetes, hyperuricemia, and the causes of nephrectomy (all *p* < 0.05) (Table 3). Patients who developed CKD stage 3 were older (62 [52–70] vs. 52 [45–61] years; *p* < 0.001) than those who did not, and had a significantly higher proportion of ASK related to nephrectomy for renal tuberculosis (44% vs. 12.3% among patients who developed CKD stage 3 vs those who did not, *p* < 0.001) (Figs. 1 and 2). The prevalence of diabetes (40.7% vs 11.8%, *p* < 0.001), hyperuricemia (46.1% vs 22.5%, *p* = 0.001), and history of CVD (34.1% vs 9.9%, *p* < 0.001) was also higher among those that developed CKD stage 3 compared to those that did not (Fig. 2). There were no significant differences with regard to sex, the location of ASK, current smoking status, and the prevalence of hypertension, dyslipidemia, and obesity between the groups. Linear regression analysis revealed a negative correlation between eGFR and the duration after nephrectomy, but the correlation did not reach statistical significance (*r =* − 0.135, *P* = 0.077) (Figs. 3).

**Table 3:** Comparison of parameters between patients who developed CKD stage 3 vs those who did not develop CKD stage 3

		Did not develop CKD stage 3	Developed CKD stage 3	Z/c^2^	P
		*n* = 81	*n* = 91		
Sex	Male	54 (66.7%)	56 (61.5%)	0.489	0.484
	Female	27 (33.3%)	35 (38.6%)		
Age (years)		52 (45–61)	62 (52–70)	−4.026	< 0.001
Duration after nephrectomy (years)		4 (3–13)	6 (2–15)	−0.947	0.344
Serum creatinine (*μmol*/*L*)		89 (72–104)	145 (117–330)	−9.761	< 0.001
eGFR (ml/min/1.73 m^2^)		81.9 ± 13.7	31.2 ± 19.7	−11.306	< 0.001
Kidney location	Left	38 (46.9%)	51 (56.0%)	1.349	0.245
	Right	43 (53.1%)	40 (44.0%)		
Cause of ASK	Tumors	47 (58%)	25 (27.5%)	25.407	< 0.001
	Tuberculosis	10 (12.3%)	40 (44%)		
	Trauma	4 (4.9%)	3 (3.3%)		
	Other	6 (7.4%)	10 (11%)		
	Unknown	14 (17.3%)	13 (14.3%)		
Current smoker	No	63 (77.8%)	69 (75.8%)	0.092	0.762
	Yes	18 (22.2%)	22 (24.2%)		
History of CVD	No	73 (90.1%)	60 (65.9%)	14.302	< 0.001
	Yes	8 (9.9%)	31 (34.1%)		
Hypertension	No	42 (52.5%)	38 (43.2%)	1.459	0.227
	Yes	38 (47.5%)	50 (56.8%)		
Obesity	No	50 (69.4%)	53 (63.9%)	0.54	0.462
	Yes	22 (30.6%)	30 (36.1%)		
Diabetes	No	67 (88.2%)	51 (59.3%)	18.847	< 0.001
	Yes	9 (11.8%)	35 (40.7%)		
Hyperuricemia	No	62 (77.5%)	48 (53.9%)	10.298	0.001
	Yes	18 (22.5%)	41 (46.1%)		
Dyslipidemia	No	54 (77.5%)	71 (88.8%)	1.411	0.235
	yes	12 (22.5%)	9 (11.2%)		

Note: Results are presented as numbers (percentages), medians (interquartile range), or medians (standard deviation); *eGFR* estimated glomerular filtration rate, *ASK* acquired solitary kidney, *CVD* cardiovascular disease

In multivariate analyses, age (odds ratio [OR] 1.076, 95% confidence interval [CI] 1.039–1.115, *p* < 0.001), diabetes (OR 4.401, 95% CI 1.693–11.44, *p* = 0.002), hyperuricemia (OR 2.733, 95% CI 1.104–6.764, *p* = 0.03), history of CVD (OR 5.583, 95% CI 1.884–18.068, *p* = 0.002), and having ASK from unilateral nephrectomy for renal tuberculosis (OR 8.816, 95% CI 2.92–26.62, *p* < 0.001) were independent risk factors for developing CKD stage 3 among the included patients with ASK (Table 4). Renal outcomes for adults with acquired solitary kidney at the end of follow-up are shown in Table 5.

**Table 4:** Risk factors associated with the development of CKD stage 3 in adults with acquired solitary kidney

Variables		B	SE	Wald	df	*P*	OR (95% CI)
Age		0.074	0.018	16.613	1	< 0.001	1.076 (1.039–1.115)
Cause of ASK	Tuberculosis	2.177	0.564	16.522 14.904	1	0.002 < 0.001	8.816 (2.92–26.62)
	Trauma	1.416	0.966	2.15	1	0.143	4.12 (0.621–27.347)
	Other	1.081	0.743	2.726	1	0.127	2.373 (0.656–12.295)
	Unknown	0.547	0.555	0.97	1	0.325	1.728 (0.582–5.129)
	Tumors	0					1
History of CVD		1.764	0.577	9.354	1	0.002	5.583 (1.884–18.068)
Diabetes		1.482	0.487	9.242	1	0.002	4.401 (1.693–11.44)
Hyperuricemia		1.005	0.462	4.727	1	0.03	2.733 (1.104–6.764)

Note: *ASK* acquired solitary kidney, *OR* odds ratio, *CI* confidence interval, *CVD* cardiovascular disease

**Table 5:** Renal outcomes for Adults with Acquired Solitary Kidney at the end of follow-up

Parameter	Value
No	172
Duration after nephrectomy (years)	5.0 (0.5–15.0)
Kidney location (L/R)	88/84
24 h urinary protein (mg)	0.44 ±0.35
Proteinuria	47 (27.3%)
Weak positive	18 (10.5%)
1+	17 (9.9%)
2+	9 (5.2%)
3+	3 (1.7%)
Hematuria	33 (19.2%)
Serum creatinine (*μmol*/*L*)	107 (87.0, 145.5)
eGFR (ml/min/1.73 m^2^)	69.1 ± 22.7
CKD	112 (65.1%)
Stage 1	7 (4.1%)
Stage 2	14 (8.1%)
Stage 3a	37 (21.5%)
Stage 3b	21 (12.2%)
Stage 4	9 (5.2%)
ESRD	24 (14.0%)
Renal replacement therapy	17 (9.9%)
Hemodialysis	15 (8.7%)
Peritoneal dialysis	2 (1.2%)
Kidney transplant	0

Note: Results are presented as numbers (percentages), medians (interquartile range), or medians (standard deviation); *eGFR* estimated glomerular filtration rate, *ASK* acquired solitary kidney, *CKD* chronic kidney disease, *ESRD* end-stage renal disease

## Discussion

In this study, we identified a high incidence of CKD stage 3 among patients with ASK due to unilateral nephrectomy for various reasons. We additionally found that higher age, a history of cardiovascular diseases, diabetes, hyperuricemia, and ASK related to renal tuberculosis were independent risk factors for subsequent CKD stage 3 in these patients. Our findings suggest that regular monitoring of renal function is needed for adult patients with ASK coupled with a history of cardiovascular diseases, diabetes, and hyperuricemia, especially among those that undergo unilateral nephrectomy for renal tuberculosis.

The prevalence of CKD stage 3 among our patients was 59.3%. Kim et al reported that the incidence of CKD among patients with solitary kidney was 2.0 per 1000 person-years [10]. Another recent study in a Chinese population reported that 25.4% patients with CSK due to unilateral renal agenesis presented with renal insufficiency during follow-up [11]. Indeed the incidence of CKD stage 3 in our cohort was higher than that observed by these studies. Several factors may be responsible for this discrepancy. First, we enrolled patients with ASK but not those with CSK. Recent studies reported that the risk of CKD in patients with ASK was significantly higher than that in patients with CSK (HR 6.18 [2.31–16.49] vs. 2.22 [0.83–5.29]), and the eGFR of those with ASK was significantly lower than that of patients with CSK [10, 12]. Second, our patients were older and a considerable proportion of them had comorbidities including diabetes and hyperuricemia, important risk factors for CKD. On the contrary, Kim et al included predominantly young to middle-aged healthy examinees and excluded those who underwent nephrectomy due to renal tuberculosis. Third, renal tuberculosis was the second most common reason for unilateral nephrectomy in our cohort, and we showed that renal tuberculosis was an independent risk factor for CKD stage 3. Furthermore, in our study, ESRD occurred in 14.0% of patients with ASK, indicating that renal outcomes can be dismal in patients with ASK. These findings are similar to those reported by Sanna-Cherchi et al regarding the renal outcomes in their study, in which 20 to 40% of patients with CSK progressed to ESRD in their thirties [13].

Although renal tumor was the most common reason for unilateral nephrectomy in our patients, the risk of CKD stage 3 in patients with ASK due to renal tumor was lower than that due to renal tuberculosis. This finding is similar to that obtained in a long-term follow-up study, which disclosed that the incidence of renal injury in children with ASK due to Wilm’s tumor was lower than that due to non-oncology reasons [14]. This may be explained by the fact that chemotherapy and radiotherapy are harmful to the remaining kidney. However, the decline of renal function associated with treatments against tumors is usually limited, reversible, and present only during the period of treatment [15].

Tuberculosis remains an important public health concern in developing countries. The incidence of tuberculosis is increasing, according to the World Health Organization, with approximately 9 million incident cases per year globally, mostly in Asia (55%) [16].

China has the third largest number of patients with tuberculosis worldwide, with an estimated incidence of 68 per 100,000 population in 2014 [17]. Urogenital tuberculosis is the third most common type of extrapulmonary tuberculosis, accounting for 27% of cases [18]. In this study, we found that renal tuberculosis was the second most common reason for unilateral nephrectomy. Moreover, the proportion of patients with ASK that received nephrectomy for renal tuberculosis was significantly higher than those with other conditions among patients who developed CKD stage 3. In the multivariate analysis, renal tuberculosis was the strongest risk factor for developing CKD stage 3 in ASK patients. Why is it that patients with ASK due to nephrectomy for renal tuberculosis are at risk for CKD stage 3? Plausible explanations are that tuberculosis involvement of the kidneys not only leads to granuloma formation in the medullary region, destroys renal parenchyma, and causes pyonephrosis and papillary necrosis, but also results in chronic interstitial nephritis and glomerular injuries such as amyloidosis [19–21]. In this sense, the remaining kidney is still susceptible to the development of CKD.

A robust association exists between CKD and cardiovascular diseases (CVDs) [22]. Patients with CKD are at increased risk for CVD, and most people with mild-to-moderate CKD die from CVD before receiving kidney replacement therapy. Conversely, CVD significantly increases the risk for morbidity and mortality in patients with CKD [23, 24]. A large, community-based study in participants free of CKD at baseline showed that the incidence rate of CKD in the heart disease cohort was 4.1 times higher than that in the comparison cohort, and that patients with heart disease are at an elevated risk of developing CKD, with an HR ranging from 3.7 to 4.99 in the older age group (age > 50 years) [25]. In our study, 39 (22.7%) patients had a history of CVD, and we similarly found that a history of CVD was an important risk factor for CKD stage 3 among patients with ASK. The pathophysiology of impaired renal function in CVD is multifactorial and includes decreased renal perfusion, atherosclerosis, inflammation, endothelial dysfunction, and neurohormonal activation [26, 27]. Therefore, patients with ASK who have a history of CVD may need close monitoring of their renal function, and optimal prevention and management of CVD may be useful for reno-protection among patients with ASK.

We discovered that higher age was an important risk factor for CKD stage 3 among patients with ASK, and that there was a trend toward a negative association between patients’ eGFR and the duration after nephrectomy. Higher age is accompanied by eGFR decrease [28], and renal aging is associated with anatomical and functional changes that accumulate over time [29]. Exposure to chronic inflammation likely enhances oxidative stress and the severity of endothelial dysfunction, both of which are related to renal aging. In addition, renal aging compromises the kidney’s ability to self-repair, and aging associated with tubular and glomerular changes further aggravates eGFR decline [29]. Therefore, elderly individuals with ASK may require close monitoring of their renal function due to their risk of subsequent CKD stage 3.

Hyperuricemia is also an independent risk factor for rapid CKD progression among children and adults [30, 31]. The presence of renal dysfunction is associated with a lower glomerular filtration rate, leading to a rise in serum uric acid and further elevating the risk of renal function decline. Animal studies have shown that uric acid can injure renal vessels in the absence of hypertension [32]. It is likely that in the presence of CKD, hyperuricemia accelerates the progression of CKD by inducing vascular damage even under optimal blood pressure control. We similarly found that hyperuricemia was an independent risk factor for CKD stage 3 among patients with ASK. However, the issue of whether the treatment of hyperuricemia would change the CKD outcomes is speculative, since it remains controversial whether hyperuricemia is a consequence or a cause (or both) of CKD [33].

Diabetic nephropathy is the most common cause of ESRD globally, and the presence of diabetes increases the risk of CKD, ESRD, and death [34]. Poor glycemic control is a risk factor for developing CKD among diabetic patients [35]. The pathogenesis of diabetic nephropathy is complex and involves glomerular hemodynamic perturbation, advanced glycation end-product formation, the generation of reactive oxygen species, and the upregulation of profibrotic growth factors such as transforming growth factors-β and connective tissue growth factors [36]. We found that diabetes was an important risk factor for CKD stage 3 among patients with ASK, with a nearly four-fold higher risk. Strict glycemic management may be useful for reno-protection among these patients with diabetes.

Hypertension is also a well-known risk factor for CKD. However, we did not find patients with CKD stage 3 having a higher prevalence of hypertension, nor was hypertension independently associated with CKD stage 3. It is possible that disparities in the regulatory pathways of fluids, electrolytes, and vasoactive hormones in those with solitary kidney account for this phenomenon. Studies with a larger sample size may be needed for confirmation. Obesity is another predisposing factor for glomerular hyperfiltration and glomerular hypertension, both of which exacerbate CKD progression [37]. A recent study involving healthy live kidney donors showed that those who were obese had 1.86 times higher risk of developing ESRD over 20 years compared to nonobese donors [38]. However, we did not find differences in the prevalence of obesity between patients who developed CKD stage 3 and those who did not. This may be explained by the low incidence of obesity among residents in Gansu province [39].

Gender difference in the risk of CKD has been documented in the general population, with men at a greater risk of developing kidney disease and having unfavorable outcomes [40]. Estrogen has been shown to have an antioxidative effect and appears reno-protective. A recent study showed that the association between solitary kidney and CKD was stronger in men [10]. However, we did not uncover any gender difference in the risk of CKD stage 3 among those with ASK, possibly related to the advanced age of our cohort (median age, 58.0 years).

Smoking is a known risk factor for many pathologies including CKD and may worsen CKD progression [41, 42]. Experimental evidence suggests that smoking contributes to endothelial dysfunction, oxidative stress, and inflammation [43]. Nicotine has been shown to worsen the severity of kidney injury in animal models of diabetes [44]. However, in this study, we failed to detect any relationship between smoking and the risk of CKD stage 3 among those with ASK. Our results were similar with those of Kim et al., whose subgroup analysis showed that smoking status had no significant interactions with the relationship between solitary kidney and CKD [10]. The reason for this phenomenon is unclear. Further research is needed to confirm our findings and to elucidate the plausible mechanisms.

Our study has several limitations. First, our study was retrospective in nature, and laboratory data were missing for some of the patients. This study could not show the negative effects of AKI on long-term renal outcomes after nephrectomy, and whether urgent or elective nephrectomy influences renal outcomes remains unclear. Second, we used the CKD-EPI formula to calculate eGFR as recommended by the Kidney Disease Outcome Quality Initiative (K/DOQI) guidelines [45]. However, the CKD-EPI formula contains limitations compared to the inulin-based GFR measurements, which is the gold standard [46]. Third, we did not record data for compensatory renal hypertrophy since renal size data were unavailable. Fourth, BP was measured manually during clinical encounters, and ambulatory blood pressure monitoring might be a better surrogate than office BP measurement for patients with solitary kidney [47]. Fifth, one of the findings in this study was the high prevalence of nephrectomy for renal tuberculosis, likely related to the high prevalence of tuberculosis in China [17]. Judging from the fact that the incidence of tuberculosis in developed countries such as those in Europe and the United States is significantly lower than in China [16], the generalizability of our findings to other populations may be limited. Finally, the single-center design and the relatively low sample size might limit the generalizability of our findings.

## Conclusions

In conclusion, adults with ASK are at risk for developing CKD stage 3, and regular follow-up of their renal function is needed, especially among those with ASK who are elderly, with diabetes, hyperuricemia, or a history of cardiovascular diseases, and those that undergo unilateral radical nephrectomy for treating renal tuberculosis. Optimal clinical management of diabetes, hyperuricemia, and cardiovascular diseases among patients with ASK may reduce their risk of developing CKD stage 3.

## Data Availability

The datasets used and/or analyzed during the current study are available from the corresponding author on reasonable request.

## Abbreviations

CKD: Chronic kidney disease
CI: Confidence interval
CSK: Congenital solitary kidney
ASK: Acquired solitary kidney
ESRD: End-stage renal disease
BMI: Body mass index
BP: Blood pressure
TC: Total cholesterol
LDL: Low-density lipoprotein
HDL: High-density lipoprotein
eGFR: Estimated glomerular filtration rate
CKD-EPI: Chronic Kidney Disease Epidemiology Collaboration
